# Children’s Interest in Digital and Traditional Literacy Activities: A Mixed-Methods Study of Parents and Children

**DOI:** 10.3390/bs16071222

**Published:** 2026-07-18

**Authors:** Galia Meoded Karabanov, Dorit Aram

**Affiliations:** The Jaime and Joan Constantiner School of Education, Tel Aviv University, Tel Aviv 6997801, Israel; dorita@tauex.tau.ac.il

**Keywords:** digital literacy, home literacy environment, preschool children, parent–child interaction, emergent literacy, digital media, early writing, early childhood

## Abstract

This mixed-methods study examined preschoolers’ digital home environment (DHE), parent–child digital and traditional literacy activities, children’s interest in literacy across modalities and parent and child perspectives on literacy practices. Participants included 121 Israeli parents of preschool-aged children and their children. Quantitative data were collected via parent questionnaires assessing joint digital literacy activities, general digital activities, parental involvement in selecting digital content, traditional literacy activities, and children’s interest in digital and traditional literacy. Qualitative data comprised parents’ open-ended responses about children’s digital media exposure and children’s perspectives on digital writing. Findings revealed positive associations between digital and traditional literacy practices in the home. Parent–child joint digital literacy activities emerged as the strongest predictor of children’s interest in digital literacy, beyond the effects of children’s age and traditional literacy practices. Conversely, parental involvement in selecting digital content was negatively associated with children’s interest in digital literacy activities. Qualitative findings indicated that parents perceived digital media use as offering educational opportunities while also raising developmental concerns, and placed strong emphasis on parental mediation and supervision. Children associated digital writing with learning, letters, and school-related literacy activities, while also linking computers with play and entertainment. Children’s preferences for handwriting versus keyboard writing were nearly equally divided, with explanations reflecting varied perceptions of convenience, enjoyment, and the meaning of writing in digital contexts. Together, these findings suggest that young children’s digital literacy experiences are shaped less by technology per se and more by the socially mediated interactions surrounding digital media use.

## 1. Introduction

Digital technologies are increasingly embedded in preschool children’s daily home lives, with many regularly using smartphones, tablets, and computers (Neumann, 2023; Soyoof et al., 2024). These technology-rich environments may support learning and school readiness (Liu et al., 2024), while also raising questions about the nature and quality of children’s interactions with digital media (Brushe et al., 2024). Within these environments, parental involvement—particularly through joint writing activities—may support children’s understanding of the writing system, highlighting parent–child digital writing as a meaningful context for early writing development (Meoded Karabanov & Aram, 2024).

Parental involvement may extend beyond regulating digital activities to encompass beliefs about their educational value. At the same time, children’s understandings of digital writing, such as typing, interacting with text, and producing written symbols, remain underexplored. Given the central role of early writing in emergent literacy (Robins et al., 2014), examining both parental perceptions and children’s representations of digital writing may provide a more comprehensive understanding of literacy development in digital home environments (Aram & Yashar, 2023).

In this mixed-methods study, we quantitatively examined preschoolers’ digital home environment (DHE), including parent–child joint digital literacy and general activities, parental involvement in selecting digital content, and children’s interest in digital literacy activities. We also examined parent–child traditional literacy activities and children’s interest in traditional literacy activities. Qualitatively, we explored parents’ perceptions of children’s digital media exposure and its developmental contributions, as well as children’s perceptions of digital writing.

### 1.1. Children’s Digital Home Environment

Most children engage with digital media at home as part of everyday literacy-related activities, such as communicating with family and friends, watching programs, playing games, and searching for information (Barr, 2019; Flewitt & Clark, 2020). For many, the home is the primary context in which they first encounter digital media and begin developing related skills, understandings, and attitudes (Marsh et al., 2017; Morgade et al., 2019). Through interactions with parents and siblings, children learn to position themselves within the digital world by imitating, exploring, and sometimes resisting digital practices as members of a digital society (Lahikainen et al., 2017).

According to Bronfenbrenner’s bioecological model (Bronfenbrenner & Morris, 2006), children’s development occurs within a sociocultural context. The home is the child’s most immediate environment, and parents, as primary mediators, play a central role in early literacy development (McBride-Chang, 2014). In line with Vygotskian theory (Vygotsky, 1978), parents scaffold their children within the Zone of Proximal Development (ZPD), whereby higher levels of parental support enable children to progress more effectively within their ZPD.

Flewitt and Clark (2020) suggest that children’s digital home environment is dynamic and shaped through meaningful human interactions that allow young children to act, intervene, and shape their own experiences. Consistent with this view, young children’s digital literacy practices at home have increased substantially in recent years. However, important aspects remain underexplored, particularly children’s perspectives, agency, creativity, and learning processes (Kumpulainen & Gillen, 2019). Most research has focused on patterns of use and parental mediation, with less attention to how children themselves experience, interpret, and actively shape their engagement with digital tools (Helsper et al., 2025; Mascheroni et al., 2018; Nikken & Schols, 2015).

Examining children’s perspectives is therefore essential for understanding the extent to which they act as agents who initiate, choose, and direct their activities, rather than simply respond to adult guidance (Barker & Weller, 2003). Exploring children’s uses and learning via digital media may also provide a more nuanced understanding of home digital literacy practices (Kumpulainen et al., 2020; Marsh et al., 2020). The present study examines preschool children’s digital literacy activities, particularly writing with parents, their interest in digital writing, and their perceptions of writing in digital contexts.

In the present study, we distinguish between three related but conceptually distinct constructs. Digital media refers broadly to children’s use of digital technologies in everyday life. Digital literacy activities refer specifically to literacy-related interactions involving digital tools, whereas digital writing refers more narrowly to writing-related activities using digital devices. Although these constructs are closely related and often overlap in children’s everyday experiences, they represent distinct concepts and are used consistently throughout this manuscript (Kazakoff, 2014).

Recent theoretical perspectives further suggest that digital literacy is not simply traditional literacy presented through a digital medium. Rather, interacting with digital technologies involves the integration of multiple processes (e.g., semantic, visuospatial, sensorimotor, executive, and socially mediated processes), highlighting that digital literacy may place partly distinct demands on learners compared with traditional literacy activities (Federico et al., 2025, 2026).

### 1.2. Parent–Child Digital and Traditional Literacy Activities

The home literacy environment (HLE) includes the availability of learning materials (e.g., books, educational games, and apps), parents’ active involvement in fostering children’s academic skills, and specific behaviors that support educational outcomes (Krijnen et al., 2020; Puranik et al., 2018; Sénéchal & LeFevre, 2014). In recent years, home literacy activities have increasingly incorporated digital elements. Researchers, educators, and parents have shown growing interest in the benefits and risks of young children’s screen exposure (Chaudron et al., 2018; Guellai et al., 2022; Ponti, 2023). The World Health Organization (2019) recommends limiting screen time for children over age two, and many parents are concerned about sedentary digital activities (Livingstone & Blum-Ross, 2020). Research further suggests that preschool children’s non-mediated screen exposure, even to educational content, offers limited benefits (Bar Lev & Elias, 2019; Barr, 2019; Korat & Segal-Drori, 2023).

Concurrently, parent–child digital literacy activities using tablets, touchscreen devices, and educational applications can support children’s literacy development, particularly when parents provide effective scaffolding (Brushe et al., 2024; Neumann, 2023; Soyoof et al., 2024; Tatar & Gerde, 2023). Such shared activities have been shown to foster emergent writing, letter knowledge, and rich language experiences (Chiong et al., 2012; Korat & Segal-Drori, 2023; Kucirkova et al., 2013; López-Escribano et al., 2021; Neumann, 2018), promote social understanding (Hiniker et al., 2018; Nathanson et al., 2013; Shachar et al., 2023), and strengthen parent–child relationships when used appropriately (Hirsh-Pasek et al., 2015). Emerging evidence suggests that well-designed digital technologies can promote children’s engagement, motivation, and participation in learning activities. Importantly, these benefits appear to depend on the quality of the learning experience and the educational design of digital activities, rather than technology use alone (Andreou & Argatzopoulou, 2024). Within the home environment, such findings further highlight the importance of examining how parent–child digital literacy activities shape children’s interest in literacy.

Alongside digital experiences, traditional parent–child literacy activities remain central to early development (Zhao et al., 2025). Such activities support language and literacy skills, including vocabulary, print awareness, phonological awareness, alphabetic knowledge, and world knowledge (Aram et al., 2013; Guo et al., 2020; Saracho, 2017). Research shows that home environments in which parents actively engage children in literacy activities promote early literacy development (Aram & Levin, 2014; Bergman Deitcher et al., 2024; Wang, 2024). Early skills such as letter knowledge and phonological awareness strongly predict later reading and writing acquisition, and differences in the home literacy environment contribute to early language and literacy gaps (Gerde et al., 2022; Nag et al., 2024; Robins et al., 2014). Parents engage children in a range of literacy activities that create a stimulus-rich environment supporting early literacy and the acquisition of reading and writing skills (Lukie et al., 2014; Piasta et al., 2012). In addition, parents’ perceptions of their role in promoting academic skills are positively associated with the frequency of home literacy activities (Sonnenschein et al., 2016).

Taken together, young children construct meaning from literacy experiences across both digital and traditional contexts (Barr, 2019), and optimal home environments integrate both domains (Chen et al., 2020). Parents’ choices of home activities reflect family cultural values that shape children’s everyday literacy experiences. For example, parents who emphasize school readiness may organize more literacy activities and be more actively involved in them (Reynolds et al., 2022). Accordingly, this study focuses on early parent–child digital and traditional literacy activities, recognizing the critical role of the early years in laying the foundation for later reading and writing development (Hegde & Mitra, 2020).

### 1.3. What We Can Learn from Parents’ Perspectives

Parents’ perspectives on young children’s use of digital media and technology reflect a complex and often contested domain within the family context. Parents frequently report tensions surrounding digital device use, including issues of access, screen time regulation, and the appropriateness of content for young children. Importantly, parents’ perspectives do not always align with children’s preferences and practices, highlighting the dynamic, negotiated nature of digital media use in early childhood (Levido et al., 2026).

These tensions reflect parents’ ongoing efforts to balance the perceived risks and benefits of digital media, particularly in relation to children’s learning and development (Wilson et al., 2025). Parental perspectives, therefore, play a key role in shaping not only children’s access to digital media but also how they use it for learning. Parents mediate children’s digital experiences by selecting content, setting boundaries, and engaging in joint activities that can support learning (Chaudron et al., 2018).

Activities involving digital technologies tend to attract children and invite independent exploration (Neumann, 2023), which further highlights the importance of parental involvement. In line with the Parental Involvement Model (Walker et al., 2005), parents’ involvement in their children’s digital world is shaped by their beliefs about what they should and can do within their children’s educational contexts. Home-based involvement often occurs when children seek assistance, and parents’ beliefs, knowledge, and availability shape the nature of that involvement.

Parental mediation practices thus determine how children encounter and use digital technologies for learning, and can be understood as a direct expression of parents’ underlying beliefs about digital media. While restrictive mediation (“gatekeeping”) may limit children’s exposure to digital learning opportunities, scaffolded co-use practices can facilitate purposeful, supported interactions with digital content. Parental perspectives, therefore, influence not only children’s access to digital media but also the purpose and educational value of their digital experiences (Dias et al., 2016). In this study, we examine parents’ perceptions of their children’s exposure to digital media and how they shape the home digital environment.

### 1.4. What We Can Learn from Children’s Perspectives

Research on children’s perspectives has increasingly shifted away from adult-centered approaches, which historically positioned children as passive subjects, toward recognizing children as active agents capable of providing meaningful insights into their own lives (Christopher, 2021). Children possess unique, experience-based knowledge of their own social worlds and are therefore important sources of data in their own right (Gallacher & Gallagher, 2008; Tsevreni et al., 2023).

Schweiger (2024) argues that children should be understood as social actors whose perspectives can enrich our understanding of childhood beyond what can be inferred from adult reports alone. This position aligns with broader developments in childhood studies that conceptualize children as “agentic” participants who interpret and shape their environments (Moore, 2015). At the same time, the literature cautions that children’s perspectives are not transparent and require careful interpretation, as they are shaped by social, cultural, and relational contexts (Spyrou, 2020). Moreover, researchers inevitably interpret children’s expressions through their own perspective, which may limit how fully children’s meanings are captured and understood (Schweiger, 2024). Consequently, incorporating children’s voices enables a more authentic and nuanced understanding of their experiences and highlights the need to critically reflect on how adults interpret children’s perspectives (Christopher, 2021; Schweiger, 2024).

This study examines children’s perceptions of writing with digital tools by using an image of a child writing on a keyboard to elicit how they see themselves as writers. From their perspective, writing is a means of expressing meaning and may include drawing, scribbling, and letter-like forms (Hall et al., 2019; Poveda, 2019). According to Vygotsky (1978), children’s development as writers is shaped through everyday interactions with print and with adults. Through these experiences, especially at home, children gradually develop their own understandings of writing and form early writing identities (Bradford & Wyse, 2013). Children’s beliefs about their own abilities also influence their writing behavior, motivation, and progress (Bandura, 1997).

Research on young children’s digital literacy provides insights into their participation in a range of digital practices, but often lacks children’s own voices, particularly regarding digital writing. For example, Marsh et al. (2017) offer a rich description of family digital literacy practices based mainly on parental reports and observations. The findings show that young children engage in various activities with digital devices and exhibit behaviors that can be interpreted as emergent forms of digital writing, such as pressing keys on a keyboard or imitating texting. Less is known about how children themselves understand or experience writing with digital tools. The present study seeks to bridge this gap by foregrounding children’s direct voices and perspectives on digital writing.

### 1.5. The Present Study

The current study examined the home digital and traditional literacy environments, children’s interest in digital and traditional literacy activities, and the perspectives of parents and children regarding digital literacy experiences. Specifically, we aimed to: (a) describe the characteristics of children’s Digital Home Environment (DHE), children’s interest in digital and traditional literacy activities, and parent–child traditional literacy activities at home; (b) examine the associations between the DHE and traditional literacy practices at home; (c) examine how demographic and background characteristics (children’s age, gender, parental education, and parent-reported independent digital device use) together with parent–child joint digital literacy activities, parent–child general digital activities, parents’ perceptions of digital tools, parents’ involvement in selecting digital content, and parent–child traditional literacy activities, explain children’s interest in digital and traditional literacy activities; (d) explore how parents perceive their children’s exposure to digital media; and (e) examine how children perceive writing with digital and traditional tools.

Based on Bioecological Systems Theory (Bronfenbrenner & Morris, 2006), Sociocultural Theory (Vygotsky, 1978), and the Parental Involvement Model (Walker et al., 2005), we hypothesized that (a) positive associations would emerge between traditional literacy practices at home and the DHE; and (b) the DHE, parents’ perceptions of digital tools, parents’ involvement in selecting digital content, and parent–child traditional literacy activities would each contribute to children’s interest in digital and traditional literacy activities beyond the effects of children’s age, gender, parental education, and parent-reported independent digital device use.

## 2. Materials and Methods

### 2.1. Study Design and Procedure

Guided by a pragmatic research paradigm (Creswell & Clark, 2017), this study used an embedded mixed-methods design. The qualitative component played a secondary role, cross-validating the quantitative findings and providing a broader perspective (Paul-Benjamin & Alpert, 2016; Schoonenboom & Johnson, 2017). The quantitative sample size (*N* = 121) exceeded the minimum recommended sample size for multiple regression analyses, as outlined in Tabachnick and Fidell (2013), supporting the adequacy of the sample for the planned analyses.

Quantitative questionnaires were used to assess the DHE, parent–child digital literacy activities, parent–child traditional literacy activities, children’s interest in traditional literacy, and children’s early writing skills. In addition, parents responded to two open-ended questions about their perceptions of children’s exposure to digital media, including their sense of control over such exposure and its perceived contribution to their child’s development. Children were interviewed and asked three open-ended questions exploring their perceptions of writing on digital devices, as well as their experiences and preferences regarding writing with digital or traditional tools. The qualitative sample was considered sufficient based on the principle of information power, as the number of responses from parents and children provided rich, relevant data that enabled the identification of recurring themes (Malterud et al., 2016).

Tel Aviv University’s Ethics Committee approved the study (No. 0009224-1). Participants were recruited through the snowball method (Baltar & Brunet, 2012). Data were collected between January and April 2025. Master’s degree students in Education approached the parents of kindergarten children and invited them to participate in the study. Data collection took place at the children’s home. Parents who agreed to participate signed a consent form and completed the study’s questionnaires. Following the parent questionnaire, each child participated in an individual interview conducted by the students, in which they responded to open-ended questions about digital writing using visual stimuli.

### 2.2. Participants

The participants were 121 parents (80.2% mothers, 19.8% fathers) and their preschool children (57.0% girls, 43.0% boys). The children’s ages ranged from 47 to 82 months (*M* = 62.22, *SD* = 8.40). The age distribution was as follows: one child aged 47 months (0.8%), 43 children aged 48–59 months (35.5%), 59 children aged 60–71 months (48.8%), and 18 children aged 72–82 months (14.9%). About half of the children were firstborn (47.8%). Most parents were married (87.1%), with an average of 2.49 children per family (*SD* = 0.99). Most parents held at least a bachelor’s degree (81.9%). In Israel, educational attainment is considered a reliable indicator of socioeconomic status (SES); therefore, the high proportion of parents with a bachelor’s degree suggests that the sample represents a middle- to high-SES population (Central Bureau of Statistics, 2020).

All participating families reported having access to digital devices at home, including mobile phones, tablets (iPads), and computers. Most families had access to multiple digital devices at home, including one to three mobile phones (85.3%), one to two tablets or iPads (60.4%), and one to two computers (72.4%). Mobile phones were the most commonly preferred devices among children (47.8%). Across devices, the most common activities included watching videos (41.7% on mobile phones; 17.2% on tablets; 9.0% on computers), playing games (16.7% on mobile phones; 15.2% on tablets; 12.6% on computers), and engaging in learning activities (7.3% on mobile phones; 10.1% on tablets; 12.6% on computers). According to parent reports, nearly half of the children (47.4%) did not use digital devices independently. Among those who did, 31.9% used digital devices independently for up to one hour per day, 15.5% for one to two hours per day, and 5.2% for two to three hours per day.

### 2.3. Measurement Instruments

#### 2.3.1. Digital Home Environment (DHE)

This 5-item self-report questionnaire assesses the DHE and young children’s digital activities at home (Meoded Karabanov & Aram, 2024). Parents were asked to rate, on a 5-point scale, how they support their children’s engagement with the digital world. Two items referred to the frequency of parent–child joint digital literacy (“How often do you play digital games involving letter recognition and reading with your child?” and “How often do you engage in writing activities with your child using digital devices (e.g., writing their name or sending a WhatsApp message together)?”); two referred to general (i.e., non-literacy-specific) digital activities (“How often do you and your child co-use digital devices at home?” and “To what extent do you explain the instructions displayed on digital devices to your child?”); one referred to parents’ involvement in selecting digital content (“To what extent are you involved in selecting the digital content your child uses?”). The average responses of parents were calculated for each measure. Higher scores indicated higher levels on the respective measure.

#### 2.3.2. Children’s Interest in Digital Literacy Activities

Parents rated their children’s interest in digital literacy activities using three items from Sonnenschein et al. (2021). Parents rated each item (e.g., “To what extent does your child show interest in writing using digital devices?”, “To what extent does your child attempt to write using digital devices e.g., on a mobile phone?”) on a 5-point scale ranging from 1 (Shows no interest) to 5 (Shows a very high level of interest). A composite score was calculated across the items.

#### 2.3.3. Parents’ Perceptions of Using Digital Devices

Parents’ perceptions of digital device use were assessed with three items from Sonnenschein et al. (2021). Parents rated each item (e.g., “To what extent does the use of digital tools contribute to the development of children your child’s age?”, “To what extent do you think it is important for preschool children to use digital devices?”) on a 5-point scale from 1 (Does not contribute at all or Not important at all) to 5 (Contributes greatly or Important to a very great extent). A composite score was calculated across items.

#### 2.3.4. Parent–Child Joint Traditional Literacy Activities at Home

This measure was adapted from Aram and Levin (2014). Parents answered three questions regarding the frequency of parent–child traditional literacy activities (e.g., “Playing letter games”, “Reading signs and labels”, and “Working with activity workbooks) on a 5-point scale from 1 (Never) to 5 (Every day). The mean score served as a composite measure.

#### 2.3.5. Children’s Interest in Traditional Literacy Activities

This measure was adapted from Martini and Sénéchal (2012) and has been used in previous studies of early literacy, including Aram and Bar-Am (2016) and Aram and Shachar (2024). In this 11-item questionnaire, parents rated their children’s interest in traditional literacy activities like writing letters, playing with letters, reading, and shared book reading (e.g., “My child enjoys writing words” and “My child enjoys copying words”) on a 5-point scale from 1 (Shows no interest) to 5 (Shows a very high level of interest). The mean score across the 11 items served as a composite measure.

#### 2.3.6. Background Measures

The parents completed a family background questionnaire, including their ages and education, the number of children in the family, and other relevant background characteristics. Parents also indicated the digital devices they have at home (mobile phone, tablet (iPad), and computer), their child’s preferred device, and the purposes for using these devices at home (e.g., watching videos, playing games, and learning activities).

### 2.4. Qualitative Measures

#### 2.4.1. Parents’ Perceptions on Children’s Exposure to Digital Media

Parents were asked to respond in writing to two optional open-ended questions examining their perceived competence and sense of control regarding their children’s use of and exposure to digital media, as well as their perceptions of the potential contribution of such exposure to children’s development. Specifically, they were asked, “To what extent do you feel confident about your child’s use of and exposure to digital media? Do you feel that the situation is under control?” and “In what ways do you believe your child’s exposure to digital devices, technologies, and media supports their development? What kinds of things does he/she learn?”

#### 2.4.2. Children’s Perceptions of Digital Writing

**Figure 1:**
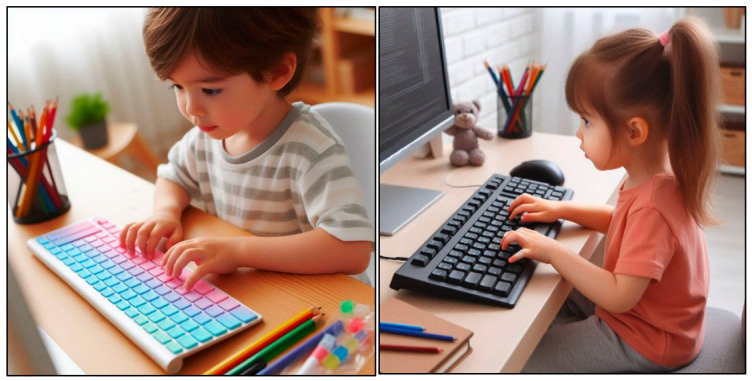
AI-generated image of a girl and a boy typing on a computer keyboard.

Children were asked to look at an image of a young girl or boy (according to their gender) sitting at a desk, typing on a computer keyboard (see Figure 1). The child was asked to respond to three open-ended questions: (1) “What is the child in the picture doing?” (2) “How do you prefer to write (pencil versus keyboard)?” and (3) “Why do you prefer writing with (the child’s preferences)?”.

The images of the girl and boy typing on a computer keyboard were generated using ChatGPT (OpenAI, 2024; GPT-4o image generation) and were used solely as a visual stimulus during the children’s interviews. They were created exclusively to elicit children’s perceptions of digital writing and to ensure that all participants responded to the same visual prompt.

### 2.5. Data Analysis

#### 2.5.1. Analysis of Quantitative Data

The selected statistical analyses were chosen to address the study’s research questions and hypotheses. Descriptive statistics were used to characterize the sample and the main study variables, including the DHE, parent–child traditional literacy activities, and children’s interest in literacy activities. Partial Pearson correlations, controlling for children’s age, were conducted to examine associations among the study variables while accounting for age-related differences in early literacy development. Hierarchical multiple regression analyses were employed to examine the unique contribution of the DHE to children’s interest in digital literacy beyond the effects of children’s demographic background and traditional home literacy activities.

#### 2.5.2. Analysis of Qualitative Data

The qualitative data were analyzed using thematic analysis, an approach well suited for identifying and interpreting recurring patterns within participants’ responses (Braun & Clarke, 2006). The analysis began with repeated holistic readings of all responses to gain an overall understanding of participants’ experiences and narratives. The first author then reread the data and generated initial inductive codes. Through constant comparison of participants’ responses, related codes were grouped into broader themes, and an initial coding framework was developed and iteratively refined to capture shared patterns and distinctive perspectives. Throughout the analysis, attention was given to both recurring patterns and unique nuances in parents’ and children’s responses. To enhance the trustworthiness and interpretive credibility of the findings, the second author reviewed the coding framework and the emerging themes, and both authors discussed coding decisions and alternative interpretations throughout the analytic process until consensus was reached. These reflexive discussions enhanced conceptual clarity and helped ensure that the themes accurately reflected the participants’ intended meanings (Creswell & Poth, 2016).

## 3. Results

### 3.1. Quantitative Findings

First, we describe DHE and traditional literacy. Descriptive statistics are presented in Table 1. Parent–child joint digital literacy activities showed a moderate mean level, as did parent–child general digital activities and their perceptions of digital tools’ contribution to children’s learning and development. In contrast, parents’ involvement in selecting digital content was relatively high. Regarding traditional literacy, parent–child joint traditional literacy activities were also moderate, while children’s interest in traditional literacy activities was relatively high. As shown in Table 1, Cronbach’s alpha coefficients for the multi-item scales in the current sample ranged from 0.67 to 0.88, indicating acceptable to good internal consistency (Nunnally & Bernstein, 1994).

Although traditional literacy activities were slightly higher than digital literacy activities, this difference was not significant, *t*(115) = −1.64, *p* = 0.104. Interestingly, children’s interest in traditional literacy activities was significantly higher than their interest in digital literacy activities, *t*(115) = −9.60, *p* < 0.001.

We studied the associations between the DHE and traditional literacy. Given the relatively wide age range of the children in the sample (47 to 82 months), child age was controlled for all correlational analyses (see Table 2).

Significant associations emerged between traditional literacy and aspects of the DHE. Parent–child joint traditional literacy activities and children’s interest in traditional literacy were positively associated with joint digital literacy activities and children’s interest in traditional literacy. Parent–child joint digital literacy activities were positively associated with general digital activities, parental involvement in selecting digital content, parents’ perceptions of digital tools, and children’s interest in digital literacy activities.

Lastly, we conducted two hierarchical regression analyses to predict children’s interest in digital and traditional literacy activities (see Table 3). In both analyses, children’s age, gender, parental education, and children’s independent digital device use were entered in the first step, and DHE measures and traditional literacy activities were entered in the second step.

Prior to conducting the hierarchical regression analyses, missing data were handled using listwise deletion, resulting in 116 complete cases for analysis. Multicollinearity was assessed using tolerance and variance inflation factor (VIF) values. Tolerance values ranged from 0.502 to 0.979, and VIF values ranged from 1.022 to 1.991, indicating no evidence of problematic multicollinearity. In addition, regression diagnostics were conducted by inspecting standardized residuals, histograms, and normal probability (P–P) plots. No substantial deviations from the assumptions of linear regression were observed.

For children’s interest in digital literacy activities, Step 1 explained 14% of the variance, with children’s age emerging as the only significant positive predictor among the control variables. In Step 2, the overall model explained 29% of the variance, a significant increase in explained variance. Parent–child joint digital literacy activities emerged as a significant positive predictor of children’s interest in digital literacy activities. In contrast, parents’ involvement in selecting digital content was negatively associated with children’s interest in digital literacy activities, such that greater parental control or monitoring of digital content was associated with lower children’s interest in digital literacy activities. Children’s gender, parental education, and children’s independent use of a digital device did not make significant unique contributions to the final model.

Although parents’ perceptions of digital tools were positively correlated with children’s interest in digital literacy, they did not make a significant unique contribution in the regression model after accounting for the other home literacy variables. This finding should be interpreted with caution, as the relatively low internal consistency of the parents’ perceptions measure may reduce the ability to detect a unique contribution. Future research should further examine the role of parents’ perceptions using measures with stronger psychometric properties.

For children’s interest in traditional literacy activities, Step 1 explained 8% of the variance, with children’s age emerging as the only significant positive predictor among the control variables. In Step 2, the overall model explained 29% of the variance, representing a significant increase in explained variance. Both parent–child joint digital literacy activities and parent–child traditional literacy activities were significant positive predictors of children’s interest in traditional literacy activities. Children’s gender, parental education, and children’s independent use of a digital device did not make significant unique contributions to the final model. These findings suggest that shared digital literacy experiences may become integrated into children’s broader literacy development, rather than replacing traditional literacy experiences.

### 3.2. Qualitative Findings

Parents were asked: “To what extent do you feel confident about your child’s use of and exposure to digital devices? Do you feel that things are under control?” A total of 98 parents (out of 121; 80.9%) provided responses. Because many parents referred to more than one aspect in their responses, individual responses were coded into multiple themes; consequently, percentages exceed 100%, and frequencies represent the number of responses per theme.

Regarding the degree of control parents felt over their children’s exposure to and use of digital media, most reported feeling in control (61%), whereas 17% reported a lack of control. An additional 21.5% reported that their children are occasionally exposed to unmonitored content and expressed concern about such exposure. We then extended this analysis by identifying themes reflecting parents’ perceptions of children’s digital media use and parental control.

Table 4 presents themes related to parents’ perceptions of children’s digital media use and parental control. Participant details presented in parentheses next to each quote indicate the parent’s or child’s gender and the child’s age.

The most prevalent theme was awareness of the importance of parental mediation and supervision, consistent with the quantitative finding of relatively high parental involvement in selecting digital content. Concerns about the negative effects of media use were also common, whereas explicit references to the educational contributions of digital media were less frequent. Child- and context-specific adaptation and the balancing of digital and non-digital activities were the least commonly represented themes. Taken together, these findings indicate ongoing tension between perceived risks and benefits, with most parents adopting a supervisory rather than permissive or prohibitive stance.

We also asked parents to report on the developmental benefits of children’s exposure to digital media. Four themes emerged (*N* = 91). The most common was academic learning (61.5%), with parents citing acquisition of letters, numbers, languages—particularly English as an additional language—and general knowledge through educational apps, videos, and digital games. Additionally, motivation, curiosity, and emotional engagement were identified in 37.4% of responses, reflecting children’s enjoyment and imaginative engagement with digital media. Digital and technological literacy appeared in 31.9% of responses, indicating that children were perceived as learning to operate devices and navigate digital environments. Notably, 7.7% of parents reported that digital media does not contribute to their child’s development and may even be harmful.

We next examined children’s perspectives. Children responded to the question “What is the child in the picture doing?” and described an image of a child typing on a computer. Six central themes emerged in their responses, reflecting their interpretations of digital writing activity (see Table 5).

The dominant theme was writing and literacy-related activities, with children attributing actions such as writing, typing, doing homework, learning, and engaging with letters and numbers to the figure in the picture. The second most frequent theme was play and entertainment, including references to playing games or watching online content. Approximately one quarter of responses were general and non-specific. Technical references to the action and associations with advanced adult-world activities each accounted for 4.1% of responses, and very few children (1.7%) reported not knowing. Children were then asked about their preferred writing tool. Preferences were nearly equally distributed: 47.9% preferred handwriting (e.g., pencil, paper, notebook) and 46.3% preferred keyboard writing. A small proportion preferred both options (1.7%), did not know or express a clear preference (2.5%), or gave responses that did not fit the main categories (1.7%; e.g., “I would rather create,” girl, 4;11). Table 6 presents themes derived from children’s explanations for their preferences.

Among children who preferred pencil writing, the most common explanation concerned ease and convenience—describing handwriting as simpler, more comfortable, or easier to use than typing on a keyboard, where one must “look for the letters” or navigate English characters. The second most frequent theme reflected a concrete conception of writing, whereby children viewed writing as a physical act inherently tied to paper, with pencil writing representing “real” writing. Affective engagement—enjoyment of handwriting—and practical access or contextual factors were less commonly cited.

Among children who preferred keyboard writing, affective engagement was most prominent, with children describing computer writing as enjoyable or expressing a general liking for digital activities. Ease and convenience was the second most common theme, with children noting that typing is faster or requires less effort than handwriting. Concrete conception of writing and access- or context-based explanations were rarely cited in this group.

### 3.3. Integration of Quantitative and Qualitative Findings

Following the separate quantitative and qualitative analyses, the findings were integrated using a joint display (McCrudden et al., 2021). This approach enabled comparison of the two data sources by identifying areas of convergence, complementarity, and expansion. The joint display of the integration summarizes how the qualitative findings complemented, explained, and extended the quantitative findings (see Table 7).

Overall, the joint display demonstrates how the qualitative findings complemented and expanded the quantitative results, providing a more comprehensive understanding of children’s digital and traditional literacy experiences.

## 4. Discussion

The present mixed-methods study examined preschool children’s Digital Home Environment (DHE), parent–child digital and traditional literacy activities, children’s interest in these activities, and the perspectives of both parents and children regarding digital writing. By integrating quantitative and qualitative findings, the study contributes to the growing literature on young children’s learning with digital technologies and expands current understanding of how digital literacy practices are embedded within family life.

Overall, the findings suggest that digital literacy activities have become integrated into young children’s everyday home experiences, yet they are deeply shaped by parental mediation, family literacy practices, and children’s own emerging understandings of writing in digital contexts. Importantly, the findings suggest that digital literacy practices do not replace traditional literacy experiences but rather coexist alongside them within the home environment.

### 4.1. Digital and Traditional Literacy as Complementary Home Practices

The first aim of the study was to examine the associations between children’s DHE and traditional literacy practices at home. The findings revealed positive associations between digital and traditional literacy practices, supporting the study’s hypothesis. These findings align with previous literature suggesting that home literacy environments are not divided into “digital” and “traditional” domains but rather reflect broader family literacy orientations and parental beliefs about learning (Barr, 2019; Chen et al., 2020; Lehrl et al., 2021). Families who engage more frequently in traditional literacy practices may also be more likely to integrate meaningful digital literacy experiences into daily routines. These findings support ecological and sociocultural perspectives, suggesting that literacy learning occurs through scaffolded interactions with more knowledgeable others (Bronfenbrenner & Morris, 2006; Vygotsky, 1978).

The qualitative findings complement this interpretation. Children frequently associated digital writing with letters, learning, and school-related activities, suggesting that they perceive digital literacy as a meaningful literacy experience rather than merely a form of technology use. These findings suggest that children view digital literacy as part of their broader literacy experiences.

Despite these positive associations, an interesting pattern emerged regarding children’s literacy interests. Although digital literacy activities were relatively common, children’s interest in traditional literacy activities was significantly higher than their interest in digital literacy activities. This finding may reflect the continuing centrality of traditional literacy experiences, such as shared book reading and handwriting, in early childhood (Hayes et al., 2026). Traditional literacy practices may provide sensory, emotional, and relational experiences that are particularly meaningful during the preschool years (e.g., Furenes et al., 2021; James, 2017). Alternatively, digital literacy activities may still represent relatively emerging practices that have not yet fully become part of children’s early literacy identities (Papadopoulou et al., 2025).

### 4.2. Parent–Child Joint Digital Literacy Activities and Children’s Interest in Digital Literacy

One of the central findings of the study was that parent–child joint digital literacy activities emerged as the strongest predictor of children’s interest in digital literacy activities, even after controlling for children’s demographic and background characteristics and traditional literacy practices. This finding reinforces growing evidence that the educational value of digital technologies in early childhood depends less on exposure itself and more on the quality of social interaction surrounding technology use (Brushe et al., 2024; Soyoof et al., 2024).

From a Vygotskian perspective, shared digital literacy activities may function as mediated learning experiences occurring within the child’s Zone of Proximal Development (ZPD), in which parents scaffold children’s participation, understanding, and engagement (Vygotsky, 1978). Through co-use practices, parents may help children interpret digital symbols, navigate literacy-related tasks, and connect digital experiences with broader linguistic and cognitive processes (Soyoof et al., 2024; Taylor et al., 2024). In this sense, digital technologies themselves do not constitute the developmental mechanism; rather, development occurs through socially mediated interactions around digital tools.

The qualitative findings further strengthen this interpretation. Many children described digital writing as involving writing, letters, learning, and school-related activities rather than merely using technology. These descriptions suggest that children perceive digital literacy as a meaningful literacy experience rather than simply a recreational activity. This qualitative evidence helps explain the quantitative finding that parent–child joint digital literacy activities were the strongest predictor of children’s interest in digital literacy. Rather than viewing digital devices primarily as entertainment, children appeared to associate them with literacy learning, likely reflecting the shared experiences they had with their parents. This is similarly supported by parents’ qualitative responses. Many parents emphasized their active role in guiding, supervising, and supporting their children’s digital experiences, suggesting that digital literacy activities often occur within a context of parental mediation. Together with the quantitative findings demonstrating frequent engagement in joint digital literacy activities, these results highlight the importance of parents not only as supervisors of technology use but also as active partners in children’s digital literacy development.

Importantly, these findings further suggest that active parental involvement, rather than passive screen exposure, is central to fostering children’s interest in digital literacy. This distinction is particularly important in light of contemporary debates surrounding young children’s screen use. Much of the public discourse continues to frame digital technologies primarily through the lens of developmental risks associated with excessive screen exposure (Ponti, 2023; World Health Organization, 2019). However, the current findings support a more nuanced perspective, according to which meaningful and scaffolded digital interactions may support children’s literacy engagement and motivation.

Interestingly, parents’ involvement in selecting digital content emerged as a small but significant negative predictor of children’s interest in digital literacy activities. One possible explanation is that highly restrictive mediation practices may reduce children’s autonomy and spontaneous engagement with digital literacy experiences. Alternatively, parents may adopt more restrictive mediation practices in response to children who already show lower interest in digital literacy activities or greater difficulties with self-regulation. This interpretation aligns with Dias et al.’s (2016) distinction between restrictive parental mediation (“gatekeeping”) and scaffolded mediation (“scaffolding”). Whereas restrictive mediation focuses primarily on limiting access, scaffolded mediation emphasizes shared exploration and guided participation. The present findings may suggest that children develop stronger engagement with digital literacy when parents participate collaboratively rather than primarily regulating access alone.

### 4.3. Exploring Parents’ Perspectives: Between Educational Opportunities and Developmental Concerns

Addressing the qualitative aim of the study, parents described complex and sometimes ambivalent perspectives regarding children’s digital media use, emphasizing both the importance of parental mediation and the educational opportunities and developmental concerns associated with digital media. This pattern aligns with the Parental Involvement Model (Walker et al., 2005), which emphasizes that parents’ beliefs regarding their educational role shape the nature and extent of their involvement in children’s learning.

Parents simultaneously highlighted academic learning, language development, technological literacy, curiosity, and motivation while expressing concerns regarding dependency, excessive screen use, attentional difficulties, and challenges in disengaging children from screens. These findings echo research describing parental tensions surrounding children’s digital media use (Livingstone & Blum-Ross, 2020; Wilson et al., 2025).

Importantly, relatively few parents completely rejected children’s exposure to digital media. Instead, most appeared to advocate a balanced approach that integrates supervision, moderation, and coexistence between digital and non-digital activities. This finding may reflect the growing normalization of digital technologies within family life and among young children. Digital media are increasingly perceived not as external additions to childhood, but as integral components of children’s everyday social and literacy environments (Livingstone & Blum-Ross, 2020).

The findings further suggest that parents do not perceive digital media as inherently positive or negative. Rather, they appear to evaluate digital experiences based on the quality of the content, parental mediation, and the balance between digital and offline activities. This view aligns with ecological perspectives that emphasize that developmental outcomes are shaped by interactions among children, technologies, and their social contexts, rather than by technology alone (Bayar et al., 2026; Goodall et al., 2025).

### 4.4. Exploring Children’s Perspectives on Digital Writing

A unique contribution of the current study is its inclusion of children’s own perspectives on digital writing, extending previous research that has relied primarily on adult reports (Christopher, 2021).

Children associated keyboard writing with literacy, learning, letters, and school-related activities, while also linking computers with play and entertainment. This pattern suggests that preschool children already recognize digital writing as a meaningful form of literacy, reflecting the hybrid nature of digital environments in early childhood.

Children’s preferences regarding handwriting versus keyboard writing were almost equally divided, indicating that both forms of writing hold meaningful places in children’s experiences. These qualitative findings complement the quantitative results by showing that children’s interest in literacy extends across both digital and traditional modalities, rather than being limited to a single form of writing.

Children who preferred handwriting often described it as more concrete, familiar, or representative of “real” writing, whereas children who preferred keyboards emphasized enjoyment, convenience, speed, and interactivity. These findings resonate with Hall et al.’s (2019) argument that young children conceptualize writing broadly and flexibly, extending beyond conventional forms.

Some children described keyboard writing as challenging because it required them to search for letters or navigate keyboards containing multiple languages, highlighting the visual and symbolic complexity of digital writing systems. In contrast, others perceived digital writing as easier and more supportive because it allowed them to correct mistakes, erase text, and produce a more accurate or “better” final product. These findings suggest that young children experience digital writing in multiple ways, shaped by their familiarity with digital tools, their fine motor skills, and their developing understandings of writing. Whereas some children perceived keyboard writing as cognitively demanding, others experienced it as a supportive tool that facilitates revision, control, and easier written expression.

Overall, the findings suggest that digital writing is experienced not only as a technical activity, but also as a meaningful social and cognitive practice embedded in children’s everyday lives. By incorporating children’s direct perspectives, the study contributes to recent calls in childhood studies to recognize children as active agents who can interpret and shape their own digital experiences (Christopher, 2021; Schweiger, 2024).

### 4.5. Implications

The findings carry educational and theoretical implications. Educationally, the study suggests that the quality of parent–child digital interactions may create conditions that are more conducive to children’s engagement in literacy activities than screen exposure alone (Hirsh-Pasek et al., 2015). Accordingly, the findings suggest that guidance for families may benefit from encouraging not only appropriate limits on digital media use but also meaningful co-use, shared digital literacy experiences, and scaffolded engagement with digital tools.

The findings also support expanding conceptualizations of the home literacy environment to include digital writing and digital literacy practices as integral components of emergent literacy. In contemporary childhood, literacy increasingly develops across multimodal environments involving print, touchscreens, keyboards, visual symbols, and interactive media.

Theoretically, the study contributes to sociocultural and bioecological perspectives on literacy development by suggesting that digital literacy practices are embedded within broader relational and family contexts. The findings further highlight the importance of integrating children’s own voices into research on digital literacy and early learning. Looking ahead, the findings seem to indicate that digital literacy may continue to play an increasingly important role in children’s home literacy environments while complementing, rather than replacing, traditional literacy practices.

### 4.6. Limitations and Future Directions

Several limitations should be acknowledged. First, the study relied primarily on parental self-report measures, which may be influenced by subjective perceptions or social desirability biases (Runge & Soellner, 2024). Because several key quantitative variables relied on parent reports, the observed associations may also have been influenced by shared-method variance (e.g., Podsakoff et al., 2024). Future research should incorporate multiple informants (e.g., teachers and children) and observational methods of parent–child interactions to reduce this potential source of bias. Nevertheless, the inclusion of qualitative data from both parents and children provided complementary perspectives that enriched the interpretation of the findings.

Second, an additional limitation concerns the visual stimuli used during the child interviews. Although the gender-matched images served solely as a visual prompt to elicit children’s perceptions of digital writing, they were not fully equivalent in their visual characteristics (e.g., keyboard appearance, surrounding objects, and body posture). These differences may have influenced children’s initial responses to the visual prompt. However, the subsequent interview questions focused on children’s own writing preferences and experiences rather than on the images themselves. Future studies should consider using fully standardized visual stimuli that differ only in the child’s gender.

Third, the sample primarily represented middle- to high-SES families with relatively high educational attainment, which may limit the generalizability of the findings across more socioeconomically and culturally diverse populations (Nag et al., 2024). Because these families generally have greater access to digital devices and educational resources, the findings may not generalize to families from lower socioeconomic backgrounds. Differences in access to technology, parental digital confidence, and opportunities for literacy-related activities may influence both children’s digital experiences and parental mediation practices (e.g., Afzal et al., 2023). Future studies should examine digital home literacy environments across broader socioeconomic and cultural contexts. Future studies should examine digital home literacy environments across more socioeconomically, culturally, linguistically, and nationally diverse populations.

Finally, although the qualitative findings provided important insight into children’s perspectives, children’s responses were relatively brief and context-dependent. Future research may benefit from incorporating additional child-centered methodologies, including observational and interaction-based approaches that examine children’s digital writing practices in more naturalistic contexts (Kumpulainen et al., 2020; Yamada-Rice, 2017). Such methods may deepen the understanding of how young children experience digital writing as part of their everyday literacy activities.

Future research should continue to examine the role of digital writing in emergent literacy development and explore how children integrate digital and traditional writing practices across home and educational contexts. Longitudinal studies may help clarify whether early parent–child digital literacy experiences contribute to later literacy outcomes, motivation, and digital competence. Additionally, future research should also examine potential gender differences in children’s digital literacy experiences, parental mediation practices, and interest in digital and traditional literacy activities.

Further research should also investigate the qualitative characteristics of parent–child digital interactions, including the types of scaffolding, communication, and emotional engagement that occur during shared digital activities. In addition, future studies may benefit from comparing different forms of digital media and writing platforms, such as tablets, keyboards, touchscreens, and AI-based educational tools.

Finally, greater attention should be given to children’s own perspectives and agency in digital literacy research. Understanding how young children themselves interpret, value, and negotiate digital writing practices may contribute to more developmentally sensitive approaches to digital literacy in early childhood education.

## 5. Conclusions

The present study demonstrates that young children’s literacy experiences increasingly develop across both traditional and digital contexts within the home environment. Parent–child joint digital literacy activities emerged as a central factor associated with children’s interest in digital literacy, highlighting the importance of socially mediated and scaffolded digital experiences. At the same time, children’s perspectives revealed that digital writing is already integrated into their emerging understandings of literacy, learning, and communication.

Together, the findings position parents as key mediators of children’s digital literacy experiences and suggest that digital writing should be understood as part of contemporary emergent literacy development rather than as separate from traditional literacy practices.

## Figures and Tables

**Table 1:** Descriptive statistics of the study measures (*N* = 121).

	Possible Range	Obtained Range	Mean (*SD*)	Cronbach’s *α*
Digital Home Environment (DHE)				
Parent–child joint digital literacy activities	1–5	1–5	2.81 (0.99)	
Parent–child general digital activities	1–5	1–5	2.67 (0.87)	
Parents’ involvement in selecting digital content	1–5	2–5	4.24 (0.88)	
Parents’ perceptions of digital tools’ contributions	1–5	1–5	2.36 (0.80)	0.67
Child’s interest in digital literacy activities	1–5	1–5	2.93 (1.12)	0.78
Traditional literacy				
Parent–child joint traditional literacy activities	1–5	1–5	2.94 (1.06)	0.73
Child’s interest in traditional literacy activities	1–5	2–5	3.89 (0.72)	0.88

**Table 2:** Correlations among the study’s measures above the diagonal and partial correlations controlling for the child’s age below the diagonal (*N* = 121).

	Digital Home Environment (DHE)					Traditional Literacy	
	1	2	3	4	5	6	7
1. Parent–child joint digital literacy activities	----						
2. Parent–child general digital activities	0.25 **	----					
3. Parents’ involvement in content selection	0.23 **	0.06	----				
4. Parents’ perceptions of digital tools	0.21 *	0.49 ***	−0.08	----			
5. Child’s interest in digital literacy activities	0.37 ***	0.27 **	−0.09	0.21 *	----		
6. Parent–child traditional literacy activities	0.62 ***	0.23 *	0.29 **	0.17	0.29 **	----	
7. Child’s interest in traditional literacy	0.36 ***	−0.05	0.10	0.01	0.34 ***	0.38 ***	----

* *p* < 0.05, ** *p* < 0.01, *** *p* < 0.001.

**Table 3:** Hierarchical Regression Analyses Predicting Children’s Interest in Digital and Traditional Literacy Activities (*N* = 121).

	Children’s Interest in			
	Digital Literacy Activities		Traditional Literacy Activities	
	(β)		(β)	
Step 1				
Children’s age	0.25 **		0.25 **	
Children’s gender	0.12		0.11	
Parental education	−0.14		−0.05	
Children’s independent digital device use	0.17		−0.12	
Step 2				
Children’s age	0.15		0.14	
Children’s gender	0.10		0.13	
Parental education	−0.10		−0.02	
Children’s independent digital device use	−0.04		−0.22	
Parent–child joint digital literacy activities	0.27 **		0.28 *	
Parent–child general digital activities	0.16		−0.13	
Parents’ perceptions of digital tools	0.02		0.06	
Parents’ involvement in selecting digital content	−0.20 *		−0.09	
Parent–child traditional literacy activities	0.15		0.29 *	
	*R* ^2^	*ΔR* ^2^	*R* ^2^	*ΔR* ^2^
Step 1	0.14 **		0.08 *	
Step 2	0.29 ***	0.15 ***	0.29 ***	0.21 ***

Note. Standardized regression coefficients (β) are reported. Unstandardized coefficients (B), standard errors (SE), 95% confidence intervals, and collinearity diagnostics were examined as part of the regression analyses. No evidence of problematic multicollinearity was found. * *p* < 0.05, ** *p* < 0.01, *** *p* < 0.001.

**Table 4:** Themes Derived from Parents’ Perceptions of Children’s Digital Media Use and Parental Control (*N* = 98).

	Theme	Example Responses	% of Responses
1	Awareness of the importance of parental mediation and supervision	“There is no use without our supervision and guidance” (mother, 4;10) “I stay next to him and choose the content together with him” (mother, 4;9)	80.6%
2	Concerns about the negative effects of media use	“There is an addiction that affects daily concentration” (mother, 6;0) “It’s very difficult to detach him from that” (mother, 5;4)	25.5%
3	Perceived contribution to learning and development	“The child gains important knowledge that will help him later in life” (father, 6;0) “Technological tools have become the main way in today’s world to consume and create content” (father, 4;11)	19.4%
4	Child- and context-specific adaptation	“Right now, it is under control because she is still young” (mother, 6;6) “There is more phone use on weekends” (mother, 5;10)	13.3%
5	Balancing between digital and non-digital activities	“There is no reason a child should grow up without screens as long as their use is limited and they are also exposed to all the activities children should engage in and play” (father, 6;3) “He mainly watches television, and the rest of the time we play and read” (mother, 4;10)	4.1%

**Table 5:** Themes for Children’s Expressions While Observing an Image of Child Typing (*N* = 121).

Question 1. “What Is the Girl or Boy in the Picture Doing?”			
	Theme	Children’s Expression	% Responses
1	Writing and literacy-related activities	“She writes on the computer” (girl, 5;11) “She’s working on letters” (girl, 4;1)	35.6%
2	Play and entertainment	“He’s watching YouTube” (boy, 5;10) “She’s playing on the computer” (girl, 5;11)	28.1%
3	General, non-specific use	“He’s doing something on the computer” (boy, 5;7) “He’s there, on the computer” (boy, 5;0)	26.4%
4	Technical reference to the action	“He presses the buttons” (boy, 5;6) “She’s on the keyboard” (girl, 4;9)	4.1%
5	Advanced adult-world activity	“She’s programming” (girl, 4;7) “She’s writing an email” (girl, 5;1)	4.1%
6	Lack of knowledge	“She’s doing… I don’t know” (girl 4;11)	1.7%

**Table 6:** Themes Derived from Children’s Explanations for their preferences (*N* = 114).

Preferred Pencil (*n* = 58; 47.9%)			
	Theme	Children’s Expression	% of Responses
1	Ease and convenience	“It’s easier, on the computer you have to look for the letters” (girl, 5;10) “Because it’s more comfortable; on the computer, there are letters in English” (girl, 5;6)	39.7%
2	Concrete conception of writing	“With a pencil you write. On the computer, you don’t” (girl, 5;3) “On paper, we can write and then show Mom and Dad” (boy, 5;6)	25.8%
3	Affective engagement	“Because it’s fun” (boy, 4;7) “I enjoy it, I have more fun writing by hand than on a keyboard” (girl, 5;11)	13.8%
4	No explanation	“I don’t know why” (girl, 5;9)	13.8%
5	Access and context	“I don’t have a computer” (girl, 6;1) “Because I’m not big enough yet [to use a computer]” (girl, 6;6)	6.9%
	Preferred keyboard (*n* = 56; 46.3%)		
1	Affective engagement	“Because you press buttons and it’s fun” (boy, 4;11) “Because I like to write things on the computer” (girl, 6;0)	41.1%
2	Ease and convenience	“Because the computer is electronic, so you can press and it writes right away… but in a notebook it takes more time” (girl, 5;10) “Because it comes out the best” (girl, 5;6)	37.5%
3	No explanation	“I don’t know why” (girl, 5;9)	14.3%
4	Access and context	“Because I have a red phone” (boy, 4;11) “Because I have a computer” (boy, 5;8)	3.6%
5	Concrete conception of writing	“Because you can write something with it” (boy, 5;5) “Because there are lots of letters there [on the keyboard]” (boy, 5;0)	3.6%

**Table 7:** Joint Display of the Integration of Quantitative and Qualitative Findings.

Quantitative Finding	Qualitative Theme	Relationship	Added Value
Digital and traditional literacy were positively associated	Parents emphasized balance between digital and traditional activities	Convergence	Supports coexistence rather than replacement
Digital literacy activities predict children’s interest	Parents described joint digital learning and mediation	Complementarity	Explains how these activities occur in everyday family life
Parental involvement negatively predicted digital literacy interest	Parents described supervision and restriction	Complementarity	Greater parental involvement may reduce children’s autonomy, or it may reflect parents’ responses to children’s lower interest or self-regulation difficulties
Children’s interest in digital literacy	Children described using digital devices as tools for writing, learning and play	Expansion	Provides children’s perspectives on the meaning of digital writing and literacy interest

## Data Availability

The data presented in this study are available on request from the corresponding author.
